# The role of the circadian timing system in sarcopenia in old age: a scoping review

**DOI:** 10.1007/s41999-024-01129-0

**Published:** 2025-01-02

**Authors:** Francesco Palmese, Ylenia Druda, Rossella Del Toro, Giorgio Bedogni, Marco Domenicali, Alessandro Silvani

**Affiliations:** 1 Department of Medical and Surgical Sciences, Alma Mater Studiorum Università di Bologna, Ravenna Campus, Ravenna, Italy; 2 Aging Research Center, Department of Neurobiology, Care Sciences and Society, Karolinska Institutet and Stockholm University, Stockholm, Sweden; 3 Department of Primary Health Care, Internal Medicine Unit Addressed to Frailty and Aging, “S. Maria Delle Croci” Hospital, AUSL Romagna, Ravenna, Italy; 4 Department of Biomedical and Neuromotor Science, Alma Mater Studiorum Università di Bologna, Ravenna Campus, Ravenna, Italy

**Keywords:** Circadian, Skeletal muscle mass, Skeletal muscle force, Physical performance, Aging

## Abstract

**Purpose:**

Sarcopenia is a progressive and generalized skeletal muscle disorder, involving the accelerated loss of skeletal muscle mass and function, associated with an increased probability of adverse outcomes including falls. The circadian timing system may be involved in molecular pathways leading to sarcopenia in older adults. We aimed to provide an updated and systematic map of the available evidence on the role of the circadian timing system in sarcopenia, specifically related to the aging process.

**Methods:**

We developed a scoping review protocol following the PRISMA-ScR guidelines. Searches were conducted on PubMed, Scopus, Web of Science,

**Results:**

We identified 373 papers from three online databases, screened 97 for full-text analysis. and selected 37 papers for inclusion. These papers included 17 primary research studies on human persons, focusing on cortisol and melatonin secretion, rest-activity rhythms, chrono-exercise, and chrono-dietary regimens, 9 primary research studies on animal models (mice, rats, fruit flies) focusing on direct expression measurement or mutations of core clock genes, and 11 narrative reviews.

**Conclusion:**

While several reports supported the role of the circadian timing system in sarcopenia, specifically related to the aging process, the available evidence is fragmented and limited. The field is thus open to preclinical and clinical research that addresses the wide knowledge gaps in the available evidence, taking advantage of what has already been published to optimize and refine experimental and clinical protocols.

**Supplementary Information:**

The online version contains supplementary material available at 10.1007/s41999-024-01129-0.

## Key summary points

**Aim:**

to provide an updated and systematic map of the available evidence on the role of the circadian timing system in sarcopenia, specifically related to the aging process.

**Findings:**

we selected 17 primary research studies on human persons, focusing on cortisol and melatonin secretion, rest-activity rhythms, chrono-exercise, and chrono-dietary regimens, 9 primary research studies on animal models (mice, rats, fruit flies) focusing on direct expression measurement or mutations of core clock genes, and 11 narrative reviews.

**Message:**

While several reports supported the role of the circadian timing system in sarcopenia, specifically related to the aging process, the available evidence is fragmented and limited. The field is open to preclinical and clinical research that should optimize research and clinical protocols to address the limitations of previously published work.

**Supplementary Information:**

The online version contains supplementary material available at 10.1007/s41999-024-01129-0.

## Introduction

Sarcopenia is a progressive and generalized skeletal muscle disorder, involving the accelerated loss of skeletal muscle mass and function, and is associated with an increased probability of adverse outcomes including falls, fractures, physical disability and mortality [1, 2]. Several operative definitions of sarcopenia have been published, including the Asian Working Group for Sarcopenia (AWGS) criteria [3], the Foundation for the National Institutes of Health Sarcopenia Project (FNIH) criteria [4], and the European Working Group on Sarcopenia in Older People (EWGSOP) criteria [5], which were revised and updated in 2019 [6]. The Global Leadership Initiative in Sarcopenia (GLIS) was launched as a collaborative effort to establish a globally accepted conceptual definition of sarcopenia and standardize commonly used terms [7, 8].

Although sarcopenia may occur at all ages and may also occur in children [9], it is especially prevalent in older adults, with important implications for public health [6].

The circadian timing system is composed of self-sustained oscillators in the suprachiasmatic nucleus of the hypothalamus (SCN), which serves as the master body clock, and in peripheral nucleated cells [10]. The SCN clock drives circadian rest-activity cycles and is entrained by the photoperiod. In turn, peripheral biological clocks are entrained by the master clock in the SCN through humoral and neuronal signals and the effects of feeding and fasting [10]. The circadian timing system modulates multiple physiological variables including the sleep–wake cycle and the secretion of hormones such as cortisol and melatonin. In the absence of time cues (Zeitgeber), the circadian timing system “free-runs” with an intrinsic period close to 24 h, whereas if the organism is exposed to and may detect the environmental Zeitgeber, the circadian timing system typically assumes the 24-h period of oscillation of ambient light. Alterations in the circadian timing system entail multiple adverse effects, including cardiometabolic risk. Taking advantage of or correcting alterations in the circadian timing system may, therefore, entail health benefits [11].

Considering the pervasiveness of the circadian timing system and the adverse consequences of its disruption, it is conceivable that this system is involved in the molecular pathways leading to sarcopenia in older adults. Due to the conservation of the circadian timing system in different organisms, important insights may be gained by integrating evidence on human persons and preclinical model systems. We reasoned that an updated and comprehensive overview of the published literature on this topic would be useful to identify knowledge gaps and help design experiments and clinical studies.

We thus aimed to provide an updated and systematic map of the available evidence on the role of the circadian timing system in sarcopenia, specifically related to the aging process. We designed and performed a scoping review to answer the following questions:

- What evidence is available on human persons?
- What evidence is available on non-human primates, rodents, invertebrates, or cell systems?
- What evidence is specifically available on muscle function, muscle mass, muscle quality (a general term broadly describing qualities of muscle beyond mass that can include histological, imaging, metabolic, or functional/impairment assessments [8]), and physical performance, as per the EWGSOP2 operative definition of sarcopenia [6]?
- What evidence addresses interactions among the circadian timing system, sarcopenia variables, and sleep, nutrition, exercise, or sex/gender?
- How much reviewed is the field, at least in terms of narrative reviews?

## Methods

### Protocol and registration

The study protocol of this review was developed following the PRISMA-ScR guidelines [12]. Although designed for systematic reviews, the PRISMA-P guidelines were also applied to this scoping review area [13]. The study protocol was deposited on the Open Science Foundation website (https://osf.io/; 10.17605/OSF.IO/TNQXR).

### Eligibility criteria

Articles were included in the study if they satisfied both of the following inclusion criteria:

- peer-reviewed research papers, letters, conference abstracts, relevant reviews, editorials, and commentaries;
- articles with explicit reference to the circadian timing system, the aging process, and either sarcopenia or at least one of the sarcopenia domains, i.e. muscle function, muscle mass, muscle quality, and physical performance.

Articles were excluded if written in languages other than English, French, German, and Italian.

### Information sources and search strategies

In March 2024, systematic electronic searches were conducted in the following databases:

- MEDLINE and PubMed Central (PMC), searched through PubMed;
- SCOPUS;
- Web of Science searched through Clarivate Analytics.

Detailed search terms for each database are available in the supplementary material as Appendix I.

### Selection of sources of evidence

After the removal of duplicate entries, the selection was performed in parallel and independently by two researchers based on title and abstract. In particular, the entries were divided into 2 groups, each of which was scored by one author with a medical geriatrics background (RDT, FP). A third author (AS) with a physiology background reviewed the entries of all groups. Disagreement was resolved by discussion of the whole author panel.

### Data charting process, data items, and synthesis of results

Data charting was performed in parallel and independently by two researchers based on a form preliminarily evaluated on a random selection of 5 entries, following the same strategy as for the selection of the sources of evidence. All variables for which data were sought are provided in the supplementary material as Appendix II.

Data extracted from the review were grouped into the following categories:

- data on human persons;
- data on non-human primates, rodents, invertebrates, or cell systems;
- data on the interactions among the circadian timing system, sarcopenia variables, and sleep, nutrition, exercise, or sex/gender;
- data on muscle function, muscle mass, muscle quality, and physical performance, as per the EWGSOP2 operative definition of sarcopenia.

A narrative synthesis approach was taken to summarize the data in each group.

The Preferred Reporting Items for Systematic Reviews and Meta-Analyses extension for Scoping Reviews (PRISMA-ScR) Checklist [12] is included as supplementary material.

## Results

### Search results

**Fig. 1:**
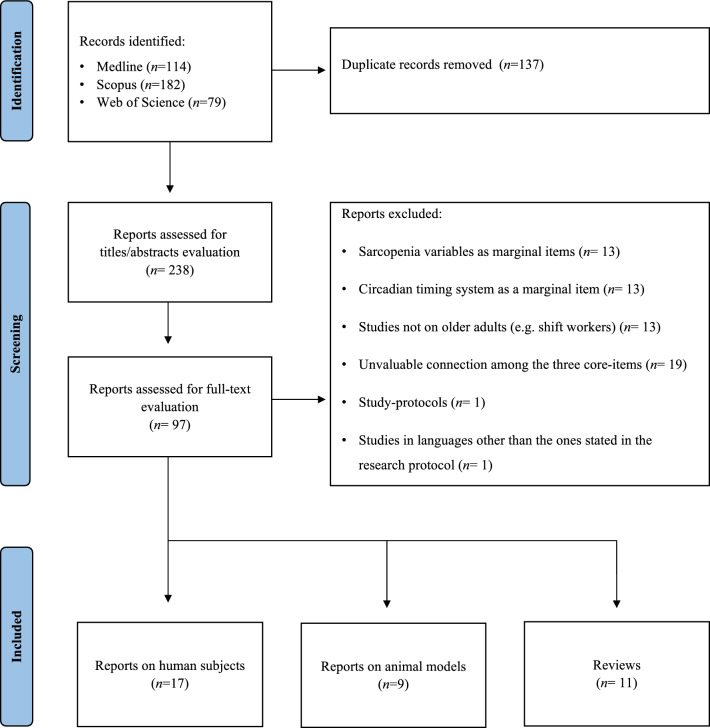
PRISMA flow diagram of the study selection process. The flow diagram was based on Moher et al. [13] according to the Preferred Reporting Items for Systematic Reviews and Meta-Analyses (PRISMA) guidelines [12]

We identified 373 articles from the online databases and screened 97 for full-text analysis. The study selection process is presented in Fig. 1.

### What evidence is available on human persons?

**Fig. 2:**
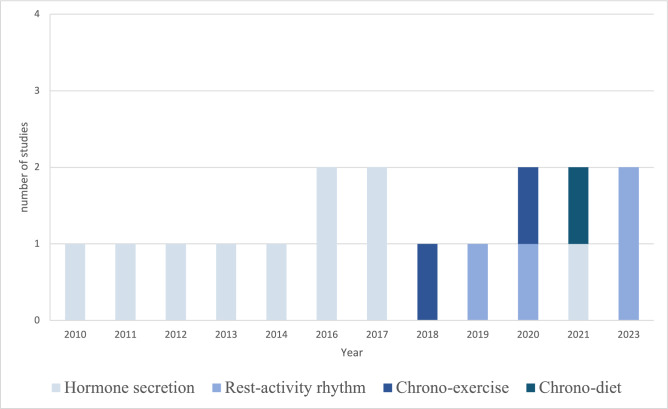
Publication dates of studies on human persons grouped according to the circadian timing system variables of interest

We selected and retrieved 17 papers on human persons. The available evidence is summarized in Table 1. To date, only four papers explicitly mentioned sarcopenia [14–17]. In the other 13 included papers, at least one of the sarcopenia variables defined by EWGSOP2 [6] was assessed. These studies mostly relied on the measurement of hormones under strong circadian control (i.e., cortisol, melatonin) as a proxy of circadian timing system function. From 2018 onwards, this approach has been complemented by the analysis of rest-activity rhythms based on wearable accelerometers and by chrono-exercise and chrono-dietary programs (Fig. 2).

**Table 1:** Studies specifically related to the aging process in human persons that addressed the role of the circadian timing system in sarcopenia variables

Authors, year	*N*	Age (years)	Sarcopenia		Circadian timing system		Main findings
			Variable	Tool/test	Variable	Tool/test	
Kumari et al., 2010 [18]	2802	60.9 (5.9)	PP	GS	Hormone secretion	SCL	Two common patterns (named “normative curve” and “raised curve”) of diurnal cortisol secretion were found. The raised pattern of secretion was associated with impaired physical function
Gardner et al., 2011 [19]	750	73.4 (4.2)	PP	TUG	Hormone secretion	SCL	Higher night-time cortisol levels were associated with slower speed and worse performance in older men. Participants with larger diurnal cortisol drops had greater speed and better performance
Heaney et al., 2012 [20]	36	72.5 (6.5)	MF	GST	Hormone secretion	SCL^a^	Lower diurnal cortisol levels were associated with lower GST
Gardner et al., 2013 [21]	2146–8448	50–92	MF PP	GST + CST WS	Hormone secretion	Serum cortisol /SCL	A larger diurnal cortisol drop was associated with faster gait speed and a quicker chair rise time in older men
Johar et al., 2014 [22]	745	65–90	MF PP	GST GS + TUG	Hormone secretion	SCL	A blunted cortisol response, lower morning, and higher evening cortisol levels were associated with frailty
Devore et al., 2016 [23]	2821	76.4 ± 5.6	MF PP	GST + CST GS	Hormone secretion	UME	No statistically significant association among UME, GST, CST, and GS in older men
Obayashi et al., 2016 [24]	760	71.1 ± 6.6	MF	GST	Hormone secretion	UME	Higher levels of UME were associated with increased GST after adjustment for potential confounders
Sousa et al., 2017 [25]	309	68.9 ± 2.8	MF PP	CST SPPB	Hormone secretion	SCL	Attenuated morning cortisol peak, higher bedtime cortisol levels, and lower morning to evening cortisol ratio were associated with worse physical performance
Dijckmans et al., 2017 [26]	60	70.6 ± 5.5	MF PP	CST GS + TUG	Hormone secretion	SCL	The variance in cortisol levels across the day from morning to evening was associated with higher GS
Krčmárová et al., 2018 [16]	31	66.6 ± 4.0	MF MM PP	CST BIA TUG	Chrono-exercise	CTP	Older women engaged in a 12-week progressive strength-training program in the morning or in the evening. The morning training group showed a relative increase in muscle mass
Maekawa and Kume, 2019 [27]	105	75.1 ± 6.1	MF PP	GST + CST GS + TUG	Rest-activity rhythm	ACT	The MSF correlated with the RA negatively in pre-frail participants and positively in non-frail participants
Lai et al., 2020 [28]	118	70.0 ± 5.0	MF PP	GST + CST GS + TUG	Chrono-exercise	ACT	Engagement in moderate-to-vigorous physical activity in the evening correlated positively with the performance at GS
Kume et al., 2020 [17]	30	76.3 ± 9.5	MF MM PP	CST BIA GS + SPPB + TUG	Rest-activity rhythm	ACT	In participants with sarcopenia, gait performance correlated negatively with IV and positively with RA and M10, whereas the skeletal muscle mass index correlated negatively with IS
Aoyama et al., 2021 [14]	60	69.6 ± 0.5	MM MF	BIA GST	Chrono-diet	N/A	Higher skeletal muscle index and grip strength were associated with higher protein consumption for breakfast than for dinner in older women without sarcopenia
Gonzalez Rodriguez et al., 2021 [15]	471	63.0 ± 7.5	MF MM	GST DXA	Hormone secretion	SCL	In postmenopausal women, sarcopenia was associated with higher salivary cortisol levels at 11 AM and 8 PM
Lee et al., 2023 [29]	340	75.4 ± 7.3	MF MM	GST BIA	Rest-activity rhythm	ACT	Especially among female participants, worse GST was associated with longer sleep duration and worse RA
Lai et al., 2023 [30]	115	70±^b^	MF PP	GST + CST GS + TUG	Rest-activity rhythm	ACT	Breaks in sedentary time (i.e., activity bouts) during the evening were associated with better gait speed, basic functional mobility and lower-limb strength

*ACT* actigraphy, *BIA* bioelectrical impedance analysis, *CST* chair stand test, *CTP* chrono-training program, *DXA* dual-energy X-ray absorptiometry, *GS* gait speed, *GST* grip strength test, *IS* inter-daily stability, *IV* intra-daily variability, *M10* activity during the most active 10-h span, *MSF* midpoint of sleep in free days, *PP* physical performance, *RA* relative amplitude, *SCL* salivary cortisol level, *MM* muscle mass, *MF* muscle function, *SPPB* short physical performance battery, *TUG* timed-up-and-go test, *UME* urinary 6-sulfatoxymelatonin excretion. Age is expressed in years as mean ± standard deviation or range depending on data available

^a^Evening and nocturnal samples were not collected (last sample at 12 h post-awakening)

^b^Standard deviation not indicated

#### Hormone secretion

Among the circadian timing system variables, hormone secretion was the most frequently evaluated, with a significant number of studies focusing on salivary cortisol levels. These studies generally agreed on the association between a higher diurnal cortisol drop (i.e., a greater difference between morning and evening cortisol concentration) and lower night-time cortisol level with better physical performance [15, 18, 19, 22, 25]. A larger diurnal cortisol drop was associated with a faster gait speed test and a quicker chair rise time also in an individual participant data metanalysis from six studies on older adults [21]. There is conflicting evidence about other parameters, especially the cortisol awakening response. This may be attributed, among other factors, to methodological differences, such as the use of a single-day protocol with one cortisol measurement [15, 18, 22] versus a more precise protocol wherein at least two samples were collected on two separate days [19].

To date, only two studies have focused on melatonin, also a hormone under strong circadian control, with discordant findings [23, 24]. In one study, among a population of 2821 older men, no statistically significant associations were found among urinary melatonin excretion levels, grip strength, gait speed, and performance at the chair stand test [23]. Conversely, in another study, higher urinary melatonin excretion was associated with higher grip strength [24].

#### Rest-activity rhythm

Three studies [17, 27, 29] investigated associations between sarcopenia variables and nonparametric metrics of the rest-activity rhythm. The first study reported that in participants with sarcopenia, gait speed correlated negatively with intra-day variability, indicating worse performance with rhythm fragmentation, and correlated positively with relative amplitude and activity in the most active 10-h span, indicating better performance with wider rest-activity rhythm and with higher daily activity levels. On the other hand, the skeletal muscle mass index correlated negatively with inter-daily stability, indicating lower muscle mass with worse synchronization to time cues [17]. In the second study, female participants with lower relative amplitude of the rest-activity rhythm were characterized by lower grip strength [29]. Conversely, no statistically significant association between grip strength or gait speed and nonparametric metrics of the rest-activity rhythm was found in a third study [27].

#### Chrono-exercise

The effects of chrono-exercise were evaluated by two studies [16, 28]. In the first study, a group of older women was divided into morning or evening study groups and engaged in a 12-week progressive strength-training program [16]. No significant differences in muscle strength and physical performance tests were observed between the groups. However, the morning training group increased muscle mass as evaluated with bioelectrical impedance analysis, suggesting that a strength training program performed in the morning may be beneficial for muscle trophism. The second study reported an association between faster gait speed and evening participation in moderate-to-vigorous physical activity in older adults (70% females) [28].

#### Chrono-dietary programs

One study [14] cross-sectionally investigated women aged 65 years or higher without sarcopenia, who were grouped according to their prevalent dietary protein assumption for breakfast or for dinner. The women who assumed more proteins for dinner than for breakfast had significantly higher skeletal muscle index and grip strength. However, causality and relevance to older participants already affected by sarcopenia are unclear due to experimental design.

### What evidence is available on non-human primates, rodents, invertebrates, or cell systems?

The available evidence on preclinical models is summarized in Table 2. We selected and retrieved nine papers on preclinical models, all of which focused on circadian clock gene expression. With the exceptions of the studies by Hunt et al. [35], on flies, and by Liang et al. [37], on rats, all studies were performed on mice.

**Table 2:** Studies specifically related to the aging process in animal models that addressed the role of the circadian timing system in sarcopenia variables

Authors, year	Model	Age (weeks)	Sarcopenia		Circadian timing system		Main findings
			Variable	Tool/test	Variable	Tool/test	
Kondratov et al., 2006 [31]	*Bmal1*-KO mice	40	MM MQ	Weight Histology	Clock genes	*Bmal1-*KO	*Bmal1*-KO mice, lacking a key component of the circadian timing system, developed age-related sarcopenia
Kondratov et al., 2009 [32]	*Bmal1*-KO mice	Up to 70	MF	GST	Clock genes	*Bmal1*-KO	Lifelong antioxidant treatment of *Bmal1*-KO mice partially prevented lifespan reduction but did not significantly prevent the decrease in grip strength
Schroder et al., 2015 [33]	iMS-*Bmal1*^−/−^ mice	70–74	MF MQ	MITF GST Histology	Clock genes	Skeletal-muscle specific, inducible *Bmal1*-KO	Inducible iMS-*Bmal1*^−/−^ mice lacking *Bmal1* selectively in skeletal muscle had decreased grip strength, skeletal muscle weakness, increased muscle fibrosis, and decreased type IIb skeletal muscle fibers
Nakao et al., 2016 [34]	MS-*Bmal1*^−/−^ mice	Up to 96	MM	Weight	Clock genes	Skeletal-muscle specific *Bmal1-*KO	The age-related decrease in skeletal muscle mass was not significantly accelerated in mice lacking *Bmal1* expression in skeletal muscles
Hunt et al., 2019 [35]	D. mel. strains with extended lifespan	Up to 14	PP MQ	Climbing, locomotion, flight, jumping Histology	Clock genes	qRT-PCR, WB	D. mel. strains selected for extended lifespan had slowed age-related decreases in physical performance and increased core clock gene expression in skeletal muscles
Nohara et al., 2019 [36]	Mice	Up to 150+	PP MF	Running wheel, treadmill GST	Clock genes	qRT-PCR, WB	Treatment with the flavonoid Nobiletine increased spontaneous wheel running activity, prolonged median survival, and increased expression of clock genes in skeletal muscles of aged mice
Liang et al., 2023 [37]	Rats	Up to 96	MM MQ	Weight Histology	Clock genes	RNA sequencing	Exercise interventions in older rats ameliorated the decrease in skeletal muscle mass and fiber cross-sectional area and led to differential expression of skeletal muscle miRNAs whose targets included circadian clock genes
Pinto et al., 2023 [38]	Mice	Up to 100 +	PP MF	Treadmill GST	Clock genes	qRT-PCR, WB	An exercise intervention in older mice ameliorated the decrease in physical performance, muscle strength, and alterations in circadian clock gene expression in the liver
Shresta et al., 2023 [39]	Mice	Up to 150	PP MF	GS GST	Clock genes	RNA sequencing	An immunotherapeutic intervention in older mice improved physical performance and muscle strength and modulated clock gene expression in the liver

Age is expressed in weeks

*D. mel.* *Drosophila melanogaster*, *GS* gait speed, *GST* grip strength test, *KO* knock-out, *MITF* maximum isometric tetanic force, *PP* physical performance, *qRT-PCR* quantitative real-time polymerase chain reaction, *MM* muscle mass, *MQ* qualities of muscle beyond mass including histological assessments, *MF* muscle function, *WB* Western blot

Four papers on mice addressed the role of the core clock gene *Bmal1* in age-related sarcopenia either at whole-body level [31, 32] or selectively at the level of skeletal muscles [33, 34]. Kondratov et al. [31] reported that *Bmal1* knock-out (KO) mice with whole-body deficiency of *Bmal1*, a core circadian clock gene, had impaired circadian timing system, decreased lifespan, and age-dependent increases in reactive oxygen species. *Bmal1*-KO mice also developed age-related decreases in skeletal muscle mass and fiber number and diameter, consistent with the development of sarcopenia. In a later study, the same group reported that lifelong antioxidant treatment of *Bmal1*-KO mice partly prevented their decrease in lifespan, but had no significant effect on their age-related decrease in grip strength [32]. These data suggest that increased oxidative stress is not an essential link between *Bmal1* expression and decreased skeletal muscle strength in mice. Schroder et al. [33] reported that a genetic mouse model lacking *Bmal1* selectively in skeletal muscles starting from adulthood had decreased skeletal muscle strength and showed muscle fibrosis and fewer glycolytic type IIb muscle fibers in old age. Conversely, Nakao et al. [34] reported that a different genetic mouse model lacking *Bmal1* selectively in skeletal muscles from conception had a skeletal muscle mass in old age that did not differ significantly from that of control animals. Overall, results on the causal role of skeletal muscle *Bmal1* in age-related sarcopenia thus appear contrasting in mice.

The association between the circadian timing system in the skeletal muscle and sarcopenia is supported by a study on Drosophila melanogaster [35]. Experimental overexpression of the core clock gene *tim* in skeletal muscles extended fly lifespan, and fly strains selected for increased lifespan had slower age-related declines in physical function and upregulation of the core clock genes *tim* and *per* in the skeletal muscles.

Four studies on older mice and rats explored the effects of exercise or pharmacological interventions on variables related to sarcopenia and on core clock genes in skeletal muscles and liver. Nohara et al. [36] found that treating old mice with a flavonoid increased the median lifespan and the peak daily spontaneous activity on a running wheel, whereas grip strength and endurance on a treadmill were not significantly affected. The flavonoid treatment also increased skeletal muscle expression of *Bmal1* and *Dec1*, a clock output gene. Liang et al. [37] reported that a range of exercise interventions in older rats increased skeletal muscle mass and fiber cross-sectional area and led to differential expression of skeletal muscle miRNAs. Functional enrichment analysis of miRNA gene targets revealed genes involved in the circadian timing system. With a similar experimental design, Pinto et al. [38] reported that an exercise intervention in older mice increased physical performance, evaluated with an incremental load test on a treadmill, and skeletal muscle strength, evaluated with a grip force test. The exercise treatment also ameliorated alterations in the expression of *Bmal1* and *Cry1* circadian clock genes in the liver. A similar conclusion is suggested by the results of Shresta et al. [39], who reported that an experimental immunotherapeutic intervention in older mice increased gait speed and grip strength and modulated the liver expression of circadian clock genes. However, the causality of the association between the circadian timing system of the liver and sarcopenia variables was not demonstrated, and the effects included both gene upregulation (*Per*, *Cry*, *Nr1d1*, *Nr1d2*, and *Dbp*) and downregulation (*Bmal1* and *Npas2*).

### What evidence is specifically available on muscle function, muscle mass, and physical performance, as per the EWGSOP2 operative definition of sarcopenia?

**Fig. 3:**
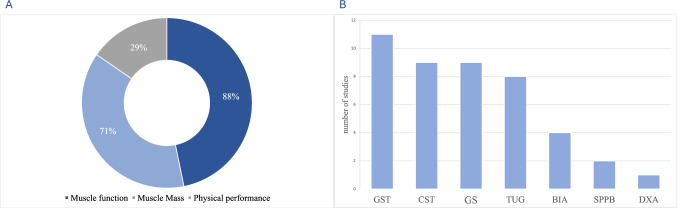
Sarcopenia variables specifically investigated by retrieved studies on the link between the circadian timing system and sarcopenia. **A** Sarcopenia variables evaluated in the selected studies; **B** Tool/test performed in the selected studies to assess sarcopenia variables. *GST* grip strength test, *CST* chair stand test, *GS* gait speed, *TUG* timed-up-and-go test, *BIA* bioelectrical impedance analysis, *SPPB* short physical performance battery, *DXA* dual-energy X-ray absorptiometry

As regards the assessment of the sarcopenia variables included in the EWGSOP2 operative definition of sarcopenia, muscle function and physical performance were the most investigated in the selected studies on human persons, as shown in Fig. 3.

Muscle mass, i.e., the “confirm” step of the diagnostic pathway of sarcopenia, were assessed only in five studies [14–17, 29], none of which employed computed tomography or magnetic resonance imaging as a specific measurement tool.

Three studies [15–17] evaluated sarcopenia more globally, without focusing on a single sarcopenia-related variable. These studies referred to different diagnostic criteria, i.e. the EWGSOP criteria [5], the EWGSOP-2 criteria [6], the Foundation for the National Institutes of Health Sarcopenia Project (FNIH) criteria [4] and the Asian Working Group for Sarcopenia (AWGS) criteria [3]. These criteria differ both in terms of cut-off values and partly in the diagnostic pathway, resulting in a different prevalence of sarcopenia in the same study population when multiple criteria are used, as in Gonzalez Rodriguez et al. [15], where both the EWGSOP-2 criteria [6] and the FNIH criteria [4] were adopted.

Surprisingly, none of the other retrieved studies on human persons explicitly referred to any sarcopenia reference values or diagnostic criteria.

In animal models, metrics of muscle mass were assessed in three studies, metrics of muscle quality specifically related to histological assessments were reported by four studies, metrics of muscle function were assessed in five studies, and metrics of physical performance were assessed in four studies.

### What evidence addresses interactions among the circadian timing system, sarcopenia variables, and sleep, nutrition, exercise, or sex/gender?

#### Sleep

Sleep was addressed in the study by Lee et al. [29], which found that longer sleep duration was associated with lower muscle mass and hand-grip strength, especially in older women. Moreover, Nohara et al. [36] showed that flavonoid treatment of older mice restored mean sleep bout duration, but not total sleep time, to levels seen in young mice. However, interactions among the circadian timing system, sarcopenia and sleep in older adults were not formally addressed by either of these studies.

#### Nutrition

Nutrition was addressed by the study by Aoyama et al. [14], which reported on the effects of greater protein intake for breakfast or for dinner in older women. However, the study lacked an independent metric of the circadian phase, precluding a meaningful analysis of interactions between the circadian timing system and nutrition.

#### Exercise

The lack of an independent metric of the circadian phase, a key parameter of the circadian timing system, also precluded a meaningful analysis of exercise-circadian interactions in studies of chrono-exercise on human persons [16, 28]. On the other hand, the studies on rodents by Liang et al. [37] and Pinto et al. [38] did include direct readouts of circadian molecular clock machinery, demonstrating that exercise regimens modulated the circadian timing system in skeletal muscles and the liver in older animals.

#### Sex or gender

Evidence specifically addressing interactions among the circadian timing system, sarcopenia variables, and sex or gender in older adults is limited, making it difficult to draw definite conclusions. Eight studies focused only on males (human persons: [19, 21, 23]; animal models: [34–38]), whereas three studies focused only on females (human persons: [14–16]). Among studies on animal models, Kondratov et al. [31] excluded sex-dependent effects only on mortality in mice, whereas effects of sex were incompletely or not reported by Kondratov et al. [32], Schroder et al. [33], and Shresta et al. [39]. Some studies on human persons simply adjusted statistically for potential confounding effects of sex [17, 22, 26–28, 30]. Other studies on human persons explicitly addressed the effects of sex/gender. In particular, Kumari et al. [18] reported that the probability of showing a raised cortisol profile was higher in men. Heaney et al. [20] did not find significant statistical effects of sex in older adults. Obayashi et al. [24] reported that overnight urinary 6-sulfatoxymelatonin excretion (UME) was higher in men; moreover, both grip strength and quadriceps muscle strength increased with UME in men, whereas only grip strength did in women. Sousa et al. [25] found stronger cortisol differences between high and low physical performance groups in women than in men but lacked the statistical power to fully examine the relevant statistical interactions. Finally, Lee et al. [29] reported associations between longer sleep duration and lower muscle mass and hand-grip strength and between lower rest-activity rhythm relative amplitude and lower hand-grip strength especially among older female participants.

### How much reviewed is the field, at least in terms of narrative reviews?

We found 11 narrative reviews mainly focusing on the molecular links between circadian timing system disruption and skeletal muscle aging [40–50]. In particular, two of these reviews were focused on hormone secretion, discussing the molecular links between melatonin and the muscular clock genes and suggesting a potential therapeutic role of melatonin against skeletal muscle atrophy [46, 50]. In another review, a hypothetical role of the nicotinamide adenine dinucleotide (NAD+) on the rejuvenation of the muscle clock was discussed, with possible therapeutic implications [49]. The therapeutic potential of chrono-nutrition against age-related muscle dysfunctions, e.g. timed-restricted feeding, was discussed in three other reviews [40, 43, 44]. However, all these hypotheses were mainly based on studies of the physiology of cellular aging, rather than on evidence on human persons. To date, no scoping review, systematic review, or meta-analysis connecting sarcopenia, the circadian timing system, and aging in human persons has been released.

## Discussion

### Summary of evidence

We performed a scoping review to provide an updated and systematic map of published evidence on the role of the circadian timing system in sarcopenia, specifically related to the aging process. We found that despite the clinical relevance of sarcopenia in older adults and the widespread interest in circadian research, published studies explicitly addressing the circadian timing system, the aging process, and sarcopenia are relatively few and have several significant limitations.

Research on human persons focused on the secretion of cortisol and melatonin, rest-activity rhythms, chrono-exercise, and chrono-dietary programs (Table 1). Cortisol and melatonin are under strong circadian control. However, other factors such as stress and sleep or ambient light modulate the secretion of cortisol and melatonin, respectively, potentially masking direct circadian control. Studies on human persons focusing on morning vs. evening exercise or dietary programs may be relevant to circadian control, as shown by studies on rodent models [37, 38], but cannot provide hard evidence in the absence of independent markers of circadian phase, a key parameter of the circadian timing system. Evidence on associations between sarcopenia and different chronotypes, i.e. morning vs. evening chronotypes, has not been widely evaluated and remains controversial [27]. Most studies on human persons were also limited by the lack of explicit operative definition of sarcopenia, by differences in the variables and tools employed to characterize muscle structure and function, and by the lack of measurement and consideration of potential confounders such as sleep, nutrition, exercise, and age/gender.

Research on animal models mainly involved mouse models, with single reports on rat and fly models. These studies had the advantage of directly addressing the circadian timing system by measuring oscillations in core clock genes or by studying organisms with mutations in core clock genes. However, core clock genes include transcription factors, such as *Bmal1*, that may also have non-circadian effects [51]. Moreover, recent data support the redundancy of the circadian timing system, with circadian rhythms persisting at the cellular level at the transcriptome, proteome, and phospho-proteome levels in the absence of *Bmal1* expression and of environmental Zeitgeber [52]. Combining the characterization of the circadian timing system at the molecular level in the skeletal muscles and/or other tissues with functional circadian timing system readouts at the organism and/or tissue level would afford a more complete assessment of circadian alterations in animal models. Moreover, the inclusion of animal models of different ages is needed for the precise characterization of the age-related development of sarcopenia.

Based on the scope and limitations of the selected studies, a proposed research agenda for further studies on the role of the circadian timing system in sarcopenia specifically related to the aging process may be suggested. The limited scope and the methodological heterogeneity of the available evidence represent significant complications with respect to performing systematic reviews and meta-analyses of published evidence at this stage. Indeed, the number of narrative reviews addressing the circadian timing system, aging and sarcopenia attests to the interest of the topic but is relatively high with respect to the published primary research. The field is, therefore, open to human and basic primary research on the role of the circadian timing system in age-related sarcopenia.

Human studies should globally evaluate sarcopenia, as opposed to its individual defining variables, and increase adherence to the international diagnostic criteria of sarcopenia. Studies with unmasking protocols such as constant routine or forced desynchrony are hardly practical in older adults with sarcopenia. However, evaluation of more than one circadian timing system variable in the same study would be desirable both in studies on humans and on animal models, particularly those involving chrono-exercise or chrono-dietary regimens, and so would the recording of environmental and in-house light exposure in studies on human persons. Finally, the study design should allow sufficient statistical power to evaluate any interaction among the circadian timing system, sarcopenia and sleep, nutrition, exercise, or sex/gender, whenever possible.

### Strengths and limitations

The points of strength of our study include a systematic approach, the use of broad eligibility criteria to minimize the exclusion of relevant studies, the search conducted on multiple databases, and the research team including authors with expertise in translational research and different backgrounds including geriatrics and physiology. A possible limitation of our study is that our inclusion criterion that the retrieved entries make explicit reference to the circadian timing system, the aging process, and sarcopenia excluded studies in which one or more of these three domains were only marginally addressed. In mitigation, this approach should have enhanced the relevance of the included studies. Another limitation is that no quality appraisal of the selected studies was performed, although this is not a specific requirement of scoping reviews.

### Conclusions

In conclusion, we provided the first systematic map of published evidence on the role of the circadian timing system in sarcopenia, specifically related to the aging process. We found several reports on human persons and animal models supporting indirectly or directly the role of the circadian timing system in sarcopenia, specifically related to the aging process. However, our scoping review revealed the relative paucity of published studies on this topic, which is potentially relevant both from the perspective of health care and from that of leveraging or correcting the circadian timing system to slow or revert age-related sarcopenia. The field is thus open to preclinical and clinical research that addresses the wide knowledge gaps in the available evidence, taking advantage of what has already been published to optimize and refine experimental and clinical protocols.

## Supplementary Information

Below is the link to the electronic supplementary material.

Supplementary file1 (DOCX 2796 KB)

Supplementary file2 (PDF 308 KB)

## Data Availability

All data relevant to the study are included in the article or uploaded as online supplemental information.
