# Supplementary material for: The role of the circadian timing system in sarcopenia in old age: a scoping review
Source: Eur Geriatr Med. 2025 Jan 2;16(2):447–60. doi: 10.1007/s41999-024-01129-0 (PMC12014836; doi:10.1007/s41999-024-01129-0)
Supplement: Supplementary file 1 — Supplementary file1 (DOCX 2796 KB) [file 41999_2024_1129_MOESM1_ESM.docx]

**Appendix I** - Search Strategy

Pubmed: "circadian"[All Fields] AND ("sarcopenia"[All Fields] OR "skeletal muscle strength"[All Fields] OR "grip strength"[All Fields] OR "chair stand test"[All Fields] OR "chair rise test"[All Fields] OR "skeletal muscle mass"[All Fields] OR "skeletal muscle quality"[All Fields] OR "skeletal muscle atrophy"[All Fields] OR "skeletal muscle cross-sectional area"[All Fields] OR "physical performance"[All Fields] OR "gait speed"[All Fields] OR "Short-physical-performance-battery"[All Fields] OR "SPPB"[All Fields] OR "Timed-up-and-go-test"[All Fields] OR "TUG"[All Fields] OR "400-meter-walk"[All Fields] OR "400-m-walk"[All Fields]) AND ("elderly"[All Fields] OR "ageing"[All Fields] OR "aging"[All Fields] OR "older adults"[All Fields] OR "middle-aged"[All Fields] OR ("middle-aged"[MeSH Terms] OR "aged"[MeSH Terms]))

Scopus: TITLE-ABS-KEY ( circadian AND ( sarcopenia OR "skeletal muscle strength" OR "grip strength" OR "chair stand test" OR "chair rise test" OR "skeletal muscle mass" OR "skeletal muscle quality" OR “skeletal muscle atrophy” OR "skeletal muscle cross-sectional area" OR "physical performance" OR "gait speed" OR "Short-physical-performance-battery" OR sppb OR "Timed-up-and-go-test" OR tug OR "400-meter-walk" OR "400-m-walk" ) AND ("elderly" OR "ageing" OR "aging" OR "older adults" OR "middle-aged"))

Web of Science: ALL = (circadian AND ( sarcopenia OR "skeletal muscle strength" OR "grip strength" OR "chair stand test" OR "chair rise test" OR "skeletal muscle mass" OR "skeletal muscle quality" OR “skeletal muscle atrophy” OR "skeletal muscle cross-sectional area" OR "physical performance" OR "gait speed" OR "Short-physical-performance-battery" OR sppb OR "Timed-up-and-go-test" OR tug OR "400-meter-walk" OR "400-m-walk" ) AND ("elderly" OR "ageing" OR "aging" OR "older adults" OR "middle-aged")).

**Appendix II** – List of variables included in the charting form

- Source (Pubmed, Scopus, Web of Science)
- Internal ID of the reference
- PMID
- DOI
- Publication Year
- Authors
- Title
- Citation
- Review (Systematic/ Scoping/ Narrative/ None)
- Editorial/commentary (Editorial/ Commentary or letter)
- Research paper/letter
- Study on a cell system (yes/no)
- Study on animal models (non-human primate, rodent, invertebrate)
- Study on humans (yes/no)
- Age
- Compliance with EWGSOP2 diagnostic criteria (yes/no, N/A)
- EWGSOP2 domain (muscle strength, muscle quantity, muscle quality, muscle performance)
- Interactions (Sleep, Nutrition, Exercise, Sex/gender)
- Main findings
